# Human cerebral vascular amyloid contains both antiparallel and parallel in-register Aβ40 fibrils

**DOI:** 10.1016/j.jbc.2021.101259

**Published:** 2021-09-29

**Authors:** Brandon A. Irizarry, Judianne Davis, Xiaoyue Zhu, Baayla D.C. Boon, Annemieke J.M. Rozemuller, William E. Van Nostrand, Steven O. Smith

**Affiliations:** 1 Center for Structural Biology, Department of Biochemistry and Cell Biology, Stony Brook University, Stony Brook, New York, USA; 2 George and Anne Ryan Institute for Neuroscience, Department of Biomedical and Pharmaceutical Sciences, University of Rhode Island, Kingston, Rhode Island, USA; 3 Department of Neurology, Alzheimer Center Amsterdam, Amsterdam Neuroscience, Amsterdam UMC - VUmc, Amsterdam, the Netherlands; 4 Department of Pathology, Alzheimer Center Amsterdam, Amsterdam Neuroscience, Amsterdam UMC - VUmc, Amsterdam, the Netherlands

**Keywords:** Alzheimer's disease, cerebral amyloid angiopathy, amyloid-β, polymorphism, antiparallel fibrils, Aβ, amyloid β, AD, Alzheimer's disease, APP, amyloid precursor protein, ATR, attenuated total reflectance, CAA, cerebral amyloid angiopathy, CAA/s, sporadic cerebral amyloid angiopathy, cryo-EM, electron cryo-microscopy, DARR, dipolar-assisted rotational resonance, ELISA, enzyme-linked immunosorbent assay, FTIR, Fourier transform infrared, LCM, laser capture microdissection, MAS, magic angle spinning, TEM, transmission electron microscopy, TBS, Tris-HCl buffered saline

## Abstract

The accumulation of fibrillar amyloid-β (Aβ) peptides alongside or within the cerebral vasculature is the hallmark of cerebral amyloid angiopathy (CAA). This condition commonly co-occurs with Alzheimer's disease (AD) and leads to cerebral microbleeds, intracranial hemorrhages, and stroke. CAA also occurs sporadically in an age-dependent fashion and can be accelerated by the presence of familial Aβ mutant peptides. Recent studies using Fourier transform infrared (FTIR) spectroscopy of vascular Aβ fibrils derived from rodents containing the double E22Q/D23N mutations indicated the presence of a novel antiparallel β-sheet structure. To address whether this structure is associated solely with the familial mutations or is a common feature of CAA, we propagated Aβ fibrils from human brain vascular tissue of patients diagnosed with nonfamilial CAA. Aβ fibrils were isolated from cerebral blood vessels using laser capture microdissection in which specific amyloid deposits were removed from thin slices of the brain tissue. Transmission electron microscopy revealed that these deposits were organized into a tight meshwork of fibrils, which FTIR measurements showed could serve as seeds to propagate the growth of Aβ40 fibrils for structural studies. Solid-state NMR measurements of the fibrils propagated from vascular amyloid showed they contained a mixture of parallel, in-register, and antiparallel β-sheet structures. The presence of fibrils with antiparallel structure derived from vascular amyloid is distinct from the typical parallel, in-register β-sheet structure that appears in fibrils derived from parenchymal amyloid in AD. These observations reveal that different microenvironments influence the structures of Aβ fibrils in the human brain.

Cerebral amyloid angiopathy (CAA) is a cerebral vascular disease primarily associated with the extracellular deposition of amyloid-β (Aβ) peptide aggregates on or within the walls of cerebral blood vessels (1, 2). In severe cases, the progressive deposition of Aβ ultimately diminishes the integrity of the affected cerebral blood vessels and can lead to smooth muscle cell degeneration, microbleeds, intracranial hemorrhages and stroke, which can all contribute to vascular cognitive impairment and dementia (3). CAA is often found in association with Alzheimer's disease (AD) in which the Aβ peptides additionally deposit in the brain parenchyma forming senile plaques (4). However, both CAA and AD can occur independently.

Several factors have been identified that influence the preferential accumulation of Aβ into either parenchymal or vascular deposits in the brain. One factor is the length of the Aβ peptide generated from the proteolytic cleavage of the amyloid precursor protein (APP). The two major cleavage products of this integral membrane protein are a 40-residue peptide (Aβ40) and a 42-residue peptide (Aβ42), accounting for ∼90% and ∼10% of the Aβ production, respectively (5, 6, 7). The more abundant Aβ40 peptide is mainly associated with amyloid deposits in the cerebral vasculature (8), which occur primarily on small cerebral arteries and arterioles in sporadic CAA (CAA/s). However, amyloid can also accumulate in capillary beds (1). In contrast, Aβ42 is the primary component in parenchymal plaques in sporadic AD (5).

A second factor influencing the location of amyloid deposition in the brain involves specific mutations within the Aβ sequence. Familial mutations at positions 22 and 23 within the Aβ region of APP are associated with enhanced vascular deposition *in vivo*. For example, the D23N (Iowa) Aβ mutation results in familial CAA characterized by amyloid deposition primarily on cerebral capillaries (1). Aβ deposition in capillary beds is often associated with severe AD (9). In contrast, the E22Q (Dutch) familial mutation results primarily in larger vessel CAA in the absence of capillary involvement (10, 11). The Dutch mutation was the first familial form of CAA to be recognized and results in early and severe cerebral vascular amyloid deposition (12, 13). Pathologically, this disorder is characterized by extensive vascular Aβ deposition, but without the key features of AD, namely cored parenchymal plaques and neurofibrillary tau pathology (10, 14, 15, 16, 17).

The molecular basis for Aβ deposition on different cerebral vascular beds is not known. However, the Aβ40-Iowa (D23N) variant has been shown to form structurally distinct antiparallel β-sheet fibrils *in vitro* compared with the parallel, in-register conformation commonly observed for wild-type Aβ40 (18). Fibrils with antiparallel β-sheet structure are stable in solution at 6 °C under quiescent conditions (18), but can convert to the parallel, in-register conformation at higher temperatures under shaking conditions (19). Similar studies regarding the Aβ40-Dutch (E22Q) variant have not yet been performed, possibly due to the inability to isolate structurally homogenous fibrils. Furthermore, antiparallel fibril character has been observed using FTIR spectroscopy of fibrils derived from vascular amyloid isolated from CAA transgenic mice (20) and rats (21), suggesting that antiparallel β-sheet structure contributes to vascular deposition. These rodent models expressed an E22Q/D23N double mutant Aβ peptide, suggesting that antiparallel Aβ structure may be solely a consequence of either or both point mutations.

An open question is whether antiparallel fibril structure occurs in nonfamilial CAA. Here, we address the structure of Aβ40-WT (wild-type) fibrils derived from cerebral blood vessel deposits extracted from human CAA tissues using negative stain transmission electron microscopy (TEM), Fourier-transformed infrared (FTIR), and solid-state NMR spectroscopy. As a control, we also investigated whether antiparallel β-sheet structure is present in Aβ40-WT fibrils derived from parenchymal amyloid deposits isolated from an atypical AD case. Vascular and parenchymal deposits derived from atypical AD cases that exhibit plaques with coarse morphology are composed mainly of the Aβ40 peptide (22). In comparison, senile plaques in typical AD are primarily composed of the Aβ42 peptide. Aβ40 fibrils derived from parenchymal amyloid in prior studies only exhibited parallel, in-register β-sheet structure (23, 24, 25).

Aβ40-WT fibrils were generated through seeded fibril growth, in which isolated amyloid deposits from diseased tissue served as a structural template for the growth of new fibrils through the addition of monomeric Aβ40-WT peptides. Our previous studies used biochemical extraction of vascular deposits by first isolating blood vessels from human brain tissue and then treating the isolated tissue with collagenase to release the embedded amyloid (20, 21). Here we introduce the use of laser capture microdissection (LCM) to isolate and study human-derived amyloid. Vascular or parenchymal amyloid deposits were first identified in thin slices of the brain tissue and then excised using laser dissection. We find using both methods that Aβ40 fibrils derived from vascular amyloid in human sporadic CAA consist of parallel and antiparallel β-sheet structures. In contrast, Aβ40 fibrils derived from parenchymal amyloid associated with atypical AD were found to contain only parallel, in-register β-sheet structure. These results are discussed in terms of the ability of the Aβ40 peptides to adopt polymorphic structures and the role of the vascular environment in influencing Aβ fibril structure.

## Results

### Isolation of vascular amyloid from patients with large vessel sporadic CAA

**Figure 1:**
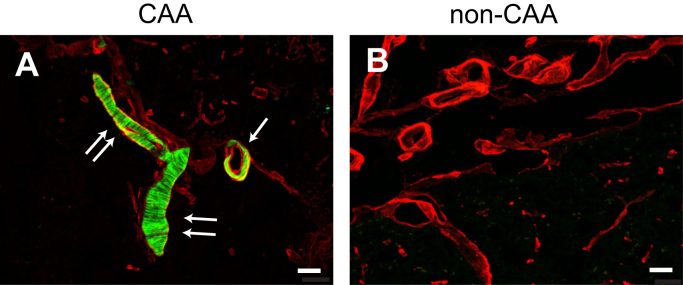
**Cerebral vascular amyloid deposition in human brain tissues.** Brain tissue sections from patients with CAA (*A*) and with no disease or amyloid plaques (non-CAA) (*B*). Thioflavin-S was used to identify vascular amyloid (*green*), and an anti-collagen IV antibody was used to identify cerebral blood vessels (*red*). The tissue slice in (*A*) shows a cross section of a blood vessel (*arrow*) and a longitudinal section of a blood vessel (*double arrows*) exhibiting amyloid buildup. In a mixture of vascular and parenchymal deposits, labeling of both the amyloid and blood vessels allows one to identify vascular amyloid in the presence of parenchymal amyloid. Scale bars = 50 μm.

Vascular amyloid deposits were isolated from a patient with AD and CAA (CAA/ad), a patient with only sporadic CAA (CAA/s), and a control patient with no vascular amyloid deposition. Both CAA cases were devoid of mutations within the Aβ peptide sequence. The CAA deposits were identified by colocalization with cerebral blood vessels (Fig. 1) using thioflavin S as a marker for amyloid fibrils (green) and antibody labeling of collagen IV as a marker for blood vessels (red). It remains unknown whether the Aβ fibril structure in vascular amyloid is similar across subtypes or patients. Here, we compare the structures of Aβ40-WT fibrils derived from vascular amyloid deposits isolated from a patient with typical AD to a second patient presenting only vascular Aβ pathology. Aβ fibrils extracted from the brain tissue containing large-vessel CAA were used as seeds to template the growth of new fibrils for structural studies (26). Two different isolation methods were compared to maximize the concentration and purity of the Aβ fibrils and to evaluate the influence of the isolation protocol on fibril morphology. The first method uses LCM, an approach that has previously not been used for isolating amyloid fibrils from the brain tissue for structural studies. As described below, it has several advantages over current methods. The second approach, which we previously employed, involves stripping intact blood vessels from the brain tissue (20, 21).

**Figure 2:**
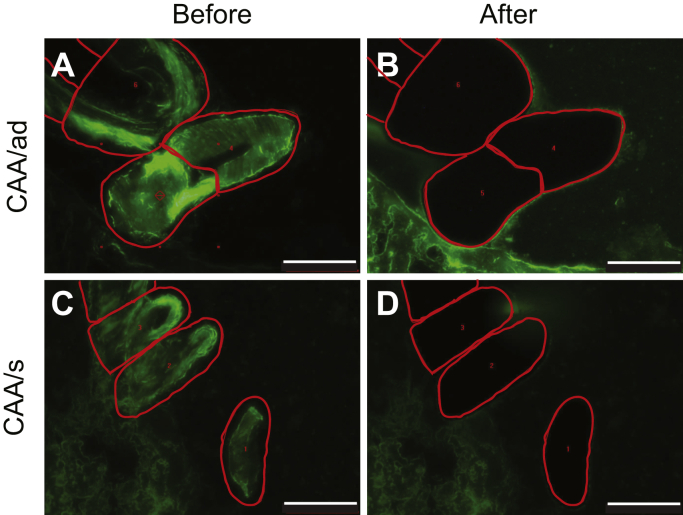
**Isolation of vascular amyloid from CAA brain by laser capture microdissection.** Fresh frozen postmortem brain tissue sections were first stained with thioflavin-S to identify vascular amyloid (*green*). Regions of interest were identified and mapped (*red traces*) prior to isolation using LCM for the CAA/ad (*A*) and CAA/s (*C*) tissue samples. Following excision, images of postdissected tissue stained with thioflavin-S (*B* and *D*) reveal the removal of the mapped regions. Scale bars are 100 μm.

In the LCM approach, brain tissue is first sectioned at 25 μm and stained with thioflavin S to identify the vascular amyloid deposits. These deposits are then collected from the tissue slices by LCM (Fig. 2, *A*–*D*). LCM has several advantages. As illustrated in the CAA/ad case, the vascular amyloid deposits can be selectively dissected from the brain tissue. The ability to isolate just the vascular amyloid deposits significantly reduces parenchymal debris compared with more conventional blood vessel isolation. The lower level of background (non-Aβ) protein present in the amyloid extract aids the use of FTIR spectroscopy to monitor Aβ40-WT fibrillization and the use of TEM to obtain images of the native fibrils following isolation. Enzyme-linked immunosorbent assays (ELISAs) of the human LCM samples showed that the vascular amyloid deposits were composed primarily (>95%) of the shorter Aβ40 peptide (data not shown).

In the second method for isolating vascular amyloid from the brain tissue, cerebral vessels are first separated from gently homogenized tissue containing large-vessel CAA amyloid deposits. Following the isolation of the cerebral vessels, the samples were treated with collagenase to aid in the dissociation of the amyloid deposits from the tissue. In Figure S1, confocal images show the isolated cerebral vessels and the extracted amyloid after collagenase treatment and purification by centrifugation, respectively. The studies below focus on the LCM method for extracting brain amyloid, but we show that the two methods yield similar results in the supporting information.

### FTIR spectroscopy as an assay of templated-fibril growth from vascular amyloid seeds

Templated fibril growth relies on the rapid conversion of monomeric Aβ into fibrils before the monomers themselves can self-nucleate. Tycko and colleagues (26) demonstrated that Aβ fibrils grown from fragmented parental fibrils (*i.e.*, seeds) produce daughter fibrils with the same morphology and molecular structure as the parental fibrils. The first step in this process is to establish a set of growth conditions that minimize the self-nucleation of Aβ40-WT monomers, while maintaining templated fibril growth. Aβ40-WT is the predominant peptide in the CAA samples, and its rate and mechanism of fibrillization are distinctly different from the Aβ42-WT peptide or the familial Aβ40 mutants (27). FTIR spectroscopy is used in the current studies to demonstrate that the resulting fibrils originate from seeded growth rather than from self-nucleation.

Temperature is a critical factor in minimizing self-nucleation of Aβ monomers. Aβ40-WT monomers fibrillize very slowly (≥48 h) at 25 °C under quiescent conditions. In contrast, under these conditions the Aβ40-WT monomer in the presence of fibril seeds can rapidly (<1 h) convert to fibrils. The amide I region (1600–1700 cm^−1^) of the FTIR spectrum is sensitive to secondary structure and allows one to follow the conversion of the sample from random coil to β-sheet structure characteristic of Aβ fibrils (28). Random coil yields a broad range of absorbances across the amide I region, while β-sheet exhibits a sharp IR band at ∼1630 cm^−1^ (29). Additionally, fibrillar maturation can be assessed by monitoring the amide II region (1500–1600 cm^−1^) for a shift from ∼1530 cm^−1^, characteristic of Aβ monomers and oligomers, to ∼1545–1550 cm ^−1^, characteristic of mature fibrils (30).

**Figure 3:**
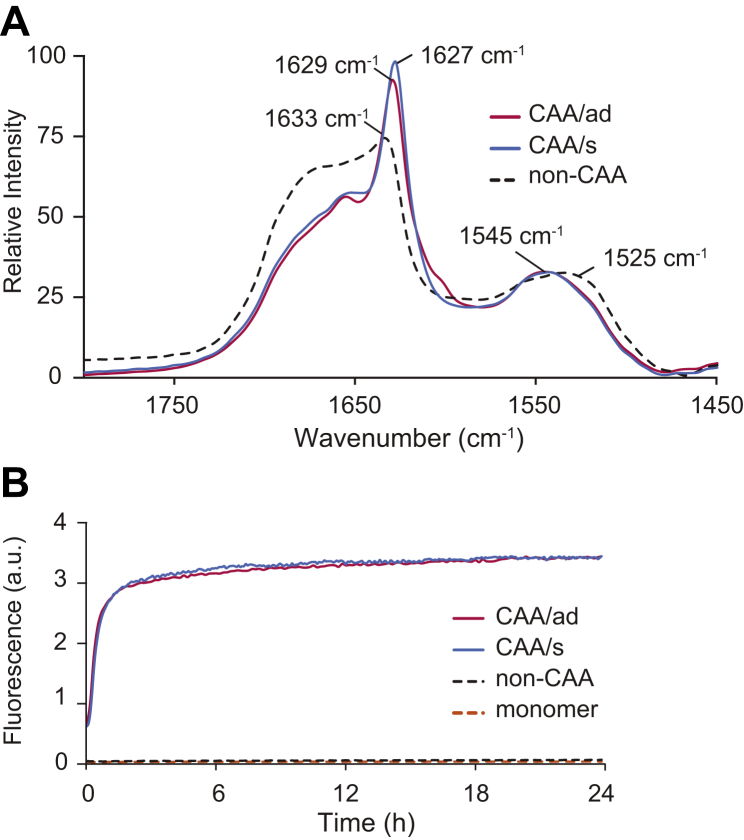
**FTIR and fluorescence spectroscopy to monitor seeded growth of fibrils from vascular amyloid seeds with Aβ40-WT monomer.***A*, FTIR spectra of the amide I and amide II regions of the Aβ40-WT monomer (100 μM) incubated in the presence of vascular amyloid isolated by LCM. The *dashed black trace* corresponds to the non-CAA control tissue incubated with Aβ40-WT monomer. The *red* and *blue traces* correspond to vascular amyloid isolated from the CAA/ad and CAA/s patients, respectively, and incubated with Aβ40-WT monomer. Spectra were obtained after 4 days of incubation at 25 °C under quiescent conditions. The presence of the amide I band at less than ∼1630 cm^−1^ and a shift of the amide II peak from ∼1530 cm^−1^ to ∼1545 cm^−1^ indicate the formation of mature Aβ40-WT fibrils. Spectra were normalized to the intensity of the amide II absorbance band. *B*, thioflavin-T fluorescence curves monitor Aβ40-WT fibril formation using fibril seeds obtained after 4 days of incubation using the samples described in (*A*). Generation-1 fibrils from the CAA/ad case (*solid red trace*) and the CAA/s case (*solid-blue trace*) result in rapid templated growth upon the addition of Aβ40-WT monomer. The non-CAA control (*dashed*, *black trace*) and Aβ40-WT monomer in the absence of seeds (*dashed*, *orange trace*) exhibit no increase in fluorescence intensity.

An overlay of FTIR spectra highlights the ability of vascular amyloid isolated from the CAA/ad and CAA/s cases to enhance the fibrillization of monomeric Aβ40-WT (Fig. 3*A*). Human brain vessels lacking vascular deposits (*i.e.*, non-CAA tissue) served as a control. These samples were isolated using LCM and incubated under quiescent conditions at 25 °C for 4 days after intermittent, low-power sonication (10-s pulses, every hour for the first 24 h). Sonication must be carefully controlled in order to minimize self-nucleation (see Figs. S2 and S3) as the tip of the sonicator probe can locally heat the sample and drive self-nucleation. The LCM sample isolated from non-CAA brain tissue after incubation with Aβ40-WT monomer (dashed black trace, Fig. 3*A*) does not exhibit an intense β-sheet band at 1629 cm^−1^ and contains a broad absorbance between 1510 cm^−1^ and 1540 cm^−1^, consistent with the presence of primarily low-molecular-weight oligomers. In contrast, the FTIR spectra corresponding to Aβ40-WT monomer incubated with vascular amyloid isolated from the CAA/ad and CAA/s LCM samples (red and blue traces, respectively) exhibit a single intense amide I band at 1627–1629 cm^−1^ and a shift in the amide II region to ∼1545 cm^−1^. These results demonstrate the successful propagation of vascular-derived Aβ40-WT fibrils from the original vascular amyloid fibrils contained in the LCM samples without contamination from monomer self-nucleation.

The amount of Aβ fibril seeds in the vascular extract is limited, resulting in low sensitivity in the FTIR measurements. The FTIR experiments performed here monitor the fibrillization of Aβ40-WT monomer in the presence of less than 1% of extracted amyloid seeds and utilize intermittent sonication to drive the samples toward maturity (see Fig. S2). Intermittent sonication expands the limited number of fibril seeds after the addition of Aβ40-WT monomer by fragmenting fibrils periodically during fibril elongation. This fragmentation promotes templated growth by expanding the number of fibril ends for solubilized monomers to attach to and drive fibril elongation. The original LCM samples are designated as generation-0 and the fibrils grown from these parent fibrils are designated as generation-1.

Additional generations of fibrils were made by taking a fraction of the preceding generation, sonicating (see Experimental procedures), and then adding monomeric Aβ peptide. To confirm that sequential generations were propagated rapidly by templated fibril growth rather than spontaneous nucleation, we made use of thioflavin-T fluorescence as well as FTIR spectroscopy. Thioflavin-T fluorescence is the conventional way to demonstrate seeded growth by comparing the rate of fluorescence increase of the Aβ monomer in the presence and absence of parental Aβ fibrils. In these experiments, the Aβ40-WT monomer alone exhibits an extended lag phase and does not transition to fibrils for more than 48 h at 25 °C under quiescent incubation conditions (dashed orange trace, Fig. 3*B*). Similarly, the Aβ40-WT monomer added to the postincubated non-CAA brain tissue sample also exhibits an extended lag phase, indicating that this tissue does not seed Aβ40-WT monomer (dashed black trace, Fig. 3*B*). In contrast, the Aβ40-WT monomer incubated with the generation-1 samples from both patients with CAA results in an immediate increase in fluorescence intensity followed by a plateau after ∼4 h (red and blue traces) demonstrating the formation of generation-2 Aβ40-WT fibrils by templated growth.

### TEM of LCM samples and seeded fibrils from sporadic CAA

**Figure 4:**
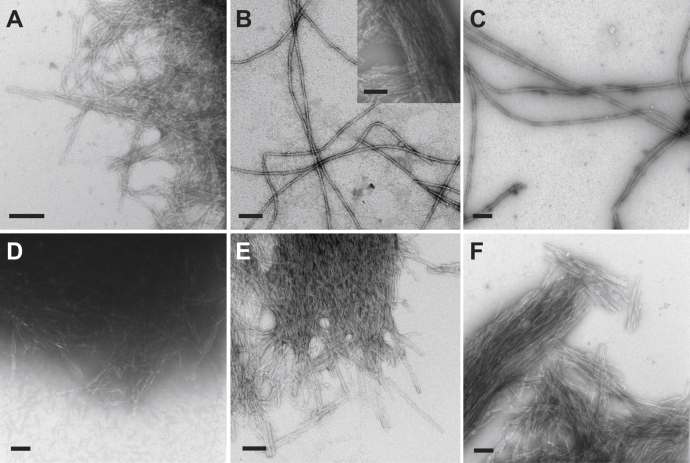
**Negative-stain TEM micrographs following the propagation of vascular-derived amyloid from human CAA/ad and CAA/s cases using LCM.***A*, vascular amyloid fibrils extracted from the CAA/ad patient using LCM (generation-0). *B*, generation-1 Aβ40-WT fibrils derived from CAA/ad vascular amyloid. *Insert* shows short, bundled fibrils that were also observed on the TEM grids. *C*, generation-3 Aβ40-WT derived from the CAA/ad patient used for NMR data acquisition in Figure 5*D*. *D*, vascular amyloid fibrils extracted from the CAA/s patient using LCM (generation-0). *E*, generation-1 Aβ40-WT fibrils derived from CAA/s vascular amyloid. *F*, generation-3 Aβ40-WT derived from the CAA/s patient used for NMR data acquisition in Figure 5*E*. Scale bars are 100 nm.

Negative-stain transmission electron microscopy (TEM) was used to visualize the native morphology of fibrils in the LCM samples. In addition, TEM was also used to assess the morphologies present in the resulting fibril samples after verification that LCM particles successfully served as seeds. TEM micrographs of vascular amyloid fibrils obtained directly after LCM isolation are shown for the CAA/ad case (Fig. 4*A*) and the CAA/s case (Fig. 4*D*). The extracted amyloid fibrils are observed along the periphery of the dense LCM particles and appear to be a tight meshwork of short fibrils with indistinguishable morphology. In the LCM method of amyloid extraction, the tissue sections and amyloid-associated proteins closely adhere to a 6–8 μm thick ethyl vinyl acetate transfer film. Neither sonication nor sequential treatment with collagenase and proteinase-K (see Experimental procedures) was found to markedly disrupt the fibril meshwork suggesting that seeded growth mainly occurs from fibrils located at the periphery of these particles.

In Figure 3, we showed that seeded growth occurs from both the CAA/ad and CAA/s LCM samples. TEM images of generation-1 Aβ40-WT fibrils derived from the CAA/ad case after incubation and intermittent sonication reveal long, isolated fibrils with a twisted morphology (Fig. 4*B*). Notably, populations of short, bundled fibrils were also observed with morphologies similar to those in the original LCM particles (*i.e.*, generation-0) (Fig. 4*B*, insert). However, these short, bundled fibrils appeared less often on the TEM grids (Fig. S4). In contrast, generation-1 fibrils derived from the CAA/s case appear as a tight network of short, bundled fibrils as seen in generation-0. Fibrils with the long, twisted morphology were only rarely observed (Fig. S4). It is important to note that monomeric Aβ40-WT did not transition into fibrils with a short, bundled morphology after incubation in the presence of preformed *in vitro* Aβ40-WT fibrils in our control experiments. Furthermore, this morphology was absent in parenchymal-derived generation-1 Aβ40-WT fibrils from an atypical AD case (see below).

The fibrils in generation-1 were propagated through two more generations using 15% parental fibrils as seeds to obtain sufficient material for NMR studies. For the CAA/ad case, the generation-3 fibrils appear morphologically similar to the long, twisted fibrils obtained in generation-1 (Fig. 4*C*). Short, bundled fibrils are observed, but are not as prominent on the TEM grids (Fig. S4). The generation-3 fibrils derived from CAA/s fibril seeds produced short, bundled fibrils similar to those observed in generation-0. The long, twisted fibrils observed in the CAA/ad case were not observed. It appears that the population of shorter, bundled fibrils is gradually replaced by the long, twisted fibrils through three generations for the CAA/ad case, while they were retained in the CAA/s case. However, we show below that the CAA/s case also results in long, twisted fibrils when seeded through six generations (Fig. S4).

### NMR spectroscopy as a probe for parallel or antiparallel Aβ40-WT fibril structure in sporadic CAA

In order to determine whether vascular-derived Aβ40 fibrils contain antiparallel fibril structure, we probed for internuclear ^13^C–^13^C distances specific to either parallel or antiparallel fibril structure using two-dimensional solid-state magic angle spinning (MAS) NMR spectroscopy. The MAS NMR approach allows one to obtain high-resolution NMR spectra of nonsoluble macromolecules (*e.g.*, amyloid fibrils) by mechanically spinning the sample at an angle of 54.7° (the magic angle) relative to the *z*-axis of the external magnetic field (31). Distance constraints, which report on fibril structure, can be obtained *via* two-dimensional dipolar recoupling methods using peptides that contain specifically ^13^C-labeled amino acids. In these experiments, the ^13^C resonances appear along the diagonal of the 2D spectrum, while off-diagonal cross-peaks between resonances appear if the ^13^C sites are separated by less than ∼6 Å. We use dipolar-assisted rotational resonance (DARR) as the 2D recoupling method for measuring internuclear distances in the experiments described below.

**Figure 5:**
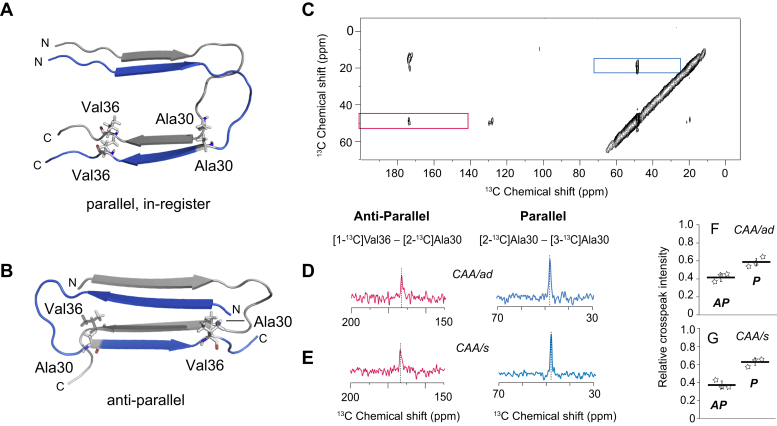
**Presence of both parallel and antiparallel Aβ40-WT fibrils in vascular-derived amyloid.***A* and *B*, fibril structures exhibiting parallel, in-register, and antiparallel β-sheet. Cross sections are shown through two cross-β units of a parallel, in-register Aβ40-WT fibril (PDB ID 2LMN) and the antiparallel Aβ40-Iowa (D23N) fibril (PDB ID 2LNQ). The fibril axis is nearly perpendicular to the plane of the page. The proximities of Ala30 and Val36 are very different in the two β-sheet geometries. In the antiparallel conformation (*B*), Ala30 and Val36 on adjacent peptides are within 6 Å of one another, and ^13^C-labels on these peptides generate cross-peaks in the 2D DARR NMR spectrum. In contrast, in the parallel, in-register conformation (*A*), these residues are >6 Å apart and interact with residues of the same residue number on the adjacent peptide. *C*, region of 2D ^13^C DARR NMR spectrum of generation-3 Aβ40-WT fibrils derived from the CAA/ad case using LCM for amyloid isolation. The diagonal NMR resonances are at ∼48 ppm for the 2-^13^C Ala30 resonance and at ∼20 ppm for the 3-^13^C Ala30 resonance. The 173 ppm diagonal resonance of 1-^13^C Val36 falls outside of the region shown. The 1-^13^C Val36 to 2-^13^C Ala30 cross-peak (*red box*) is consistent with antiparallel β-sheet structure. The 2-^13^C Ala30 to 3-^13^C Ala30 cross-peak (*blue box*) is consistent with parallel, in-register β-sheet structure. *D* and *E*, rows are shown through the diagonal resonances boxed in panel (*C*) of generation-3 Aβ40-WT fibrils derived from the CAA/ad (*D*) and CAA/s (*E*) LCM samples. *F* and *G*, relative cross-peak intensities of generation three fibrils. Three independent experiments (n = 3) were carried out for the CAA/ad case (*D*) and for the CAA/s case (*E*) to establish the reproducibility of the experiments from LCM isolation to the NMR measurements. The total intensity for the boxed regions in panel (*C*) corresponding to the antiparallel (*red*, AP) and parallel, in-register (*blue*, P) cross-peaks was normalized to 1. The relative intensities for both parallel and antiparallel cross-peaks were significant (*p* < 0.01) relative to the noise using a Student's paired *t* test with a two-tailed distribution.

For the 2D DARR MAS NMR experiments, we used two Aβ40-WT peptides with different ^13^C labels in a 1:1 ratio to simultaneously assess the presence of either parallel or antiparallel fibril structure within the same sample (19) (Fig. 5, *A* and *B*). The ^13^C-labeled Aβ40-WT monomeric peptides were added to seeds (15% w/w) derived from generation-2 Aβ40-WT fibrils corresponding to the CAA/ad and CAA/s cases. Seeded samples were incubated at 25 °C under quiescent conditions and were shown to convert to mature generation-3 fibrils using FTIR spectroscopy prior to NMR measurements.

NMR measurements were made of generation-3 fibrils from both the CAA/ad and CAA/s cases. A region of the 2D ^13^C NMR spectrum of generation-3 Aβ40-WT fibrils for the CAA/ad case containing cross-peaks diagnostic of parallel, in-register, and antiparallel fibril β-sheet structure is shown in Figure 5*C*. The NMR resonances along the diagonal in the 2D spectrum correspond to resonances observed in a 1D ^13^C NMR spectrum, while the off-diagonal cross-peaks arise from ^13^C dipolar couplings between residue-specific ^13^C sites that are within ∼6 Å of each other. The region boxed in red displays a cross-peak resulting from dipolar couplings between 2-^13^C Ala30 and 1-^13^C Val36 on adjacent β-strands, which is consistent with the antiparallel Aβ40-Iowa fibril structure where these sites are ∼4.5 Å apart (18). The region boxed in blue exhibits a cross-peak resulting from dipolar couplings between 2-^13^C Ala30 and 3-^13^C Ala30 on separate peptides, which is consistent with parallel, in-register Aβ fibrils where these sites are ∼4.5 Å apart.

Rows taken through the diagonal resonances of 2-^13^C Ala30 and 3-^13^C Ala30 better illustrate the cross-peaks associated with antiparallel structure (red) and parallel, in-register structure (blue) for each sample (Fig. 5, *D* and *E*). The Aβ40-WT generation-3 fibrils derived from both the CAA/ad and CAA/s deposits exhibit cross-peaks between 1-^13^C Val36 and 2-^13^C Ala30 (red trace), as well as cross-peaks between 2-^13^C Ala30 and 3-^13^C Ala30 (blue trace), indicating the coexistence of antiparallel and parallel, in-register fibril structure, respectively. In order to demonstrate the reproducibility of the NMR measurements, three independent experiments were undertaken on both the CAA/s and CAA/ad cases starting from new vascular amyloid samples collected by LCM. To quantify the relative population of each structural form, the total intensity for regions containing cross-peaks consistent with antiparallel and parallel, in-register fibril structures was normalized to 1. A ratio of the intensity for cross-peaks consistent with antiparallel or parallel, in-register fibril structure to the total intensity (sum of all cross-peak intensities within each sample) showed only a small variation between samples (Fig. 5, *F* and *G*).

Generation-3 fibrils were similarly formed using vascular amyloid seeds from the CAA/ad and CAA/s cases using the stripped vessel isolation method. NMR measurements on these fibrils using the same ^13^C-labeling scheme as the LCM-derived fibrils exhibited both antiparallel and parallel cross-peaks (Fig. S1). A comparison between the NMR results using the two amyloid isolation methods indicates that the approach used does not influence the seeded fibril structure.

Two sets of control experiments were undertaken to demonstrate that antiparallel fibrils are not observed unless they are present in the parental fibril seeds under the seeded growth conditions used here. In the first control experiment, Aβ40-WT fibrils were grown *in vitro* and shown by solid-state NMR to contain only the parallel, in-register conformation. When used as seeds, these fibrils replicated the parallel, in-register conformation without the presence of antiparallel specific NMR cross-peaks (Fig. S6).

In the second control experiment, parenchymal amyloid plaques isolated using LCM from a patient exhibiting atypical AD, but lacking vascular amyloid deposits, were used to seed fibril growth. ELISAs of the atypical AD plaques indicated that they contain predominantly Aβ40 (22). As a result, we were able to use Aβ40-WT monomer to grow fibrils from the parenchymal LCM samples using the same protocol as described for the vascular LCM samples. NMR measurements of generation-3 atypical AD fibrils revealed only parallel, in-register β-sheet structure (Fig. S7). Moreover, TEM images of the generation-1 parenchymal-derived fibrils lack the short, bundled morphology. Only long, twisted fibrils are observed. These results agree with other studies on brain-derived fibrils obtained from parenchymal seeds showing that parenchymal plaques are composed of fibrils containing parallel, in-register β-sheet structure (23, 32) and suggest that antiparallel β-sheet fibril structure is unique to vascular amyloid deposits.

### Stability of the antiparallel fibril conformation

The solid-state NMR measurements indicate that antiparallel and parallel, in-register Aβ fibril structures contribute to vascular amyloid in patients with nonfamilial CAA. In the case of the Aβ40-Iowa peptide, *in vitro* studies by Tycko and colleagues (33) showed that this peptide can form both conformations. The antiparallel conformation could be isolated by filtration (18), whereas the parallel, in-register conformation can be selected by rapidly seeding through multiple generations (34). In this section, we seeded through multiple generations to determine if the antiparallel structure is persistent or whether the different polymorphs have different seeding efficiencies and a single defined structure can be isolated.

We propagated generation-3 fibrils to generation-6 using 5% (w/w) of parental fibril seeds. The lower percentage of seeds exploits the potential for differential seeding efficiencies between parallel and antiparallel Aβ40-WT fibrils. We first show that the short, bundled fibrils in generation-1 give way to elongated twisted, fibrils upon continued propagation (Fig. S4).

**Figure 6:**
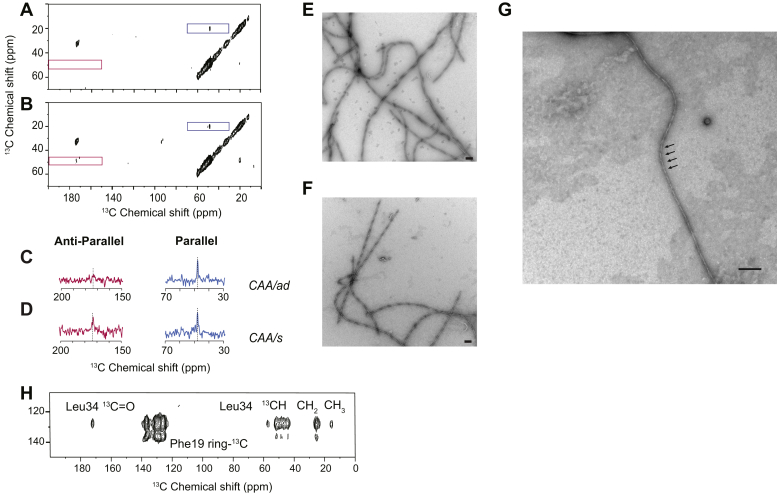
**Propagation of generation-3 fibrils to generation-6.***A* and *B*, region of 2D ^13^C solid-state DARR NMR spectra of generation-6 Aβ40-WT fibrils derived from the CAA/ad (*A*) and CAA/s (*B*) cases. The region of the spectrum exhibiting cross-peaks consistent with parallel, in-register β-sheet structure (*blue box*) and antiparallel β-sheet structure (*red box*) are shown. *C* and *D*, rows are shown through the diagonal resonances of 2-^13^C Ala30 at 48.6 ppm (*red*) and 3-^13^C Ala30 at 20.1 ppm (*blue*) of generation-6 Aβ40-WT fibrils derived from the CAA/ad (*C*) and CAA/s (*D*) cases. *E–G*, TEM images of generation-6 Aβ40-WT fibrils from the CAA/ad (*E* and *G*) and CAA/s (*F*) cases. The predominant morphology is twisted fibrils with crossing points of ∼100–200 nm. However, highly twisted fibrils with crossing points of <50 nm were also observed. In panel (*G*), *arrows* indicate representative crossing points with a spacing of <50 nm. Scale bars are 100 nm. *H*, Region of the 2D ^13^C DARR NMR spectrum of generation-6 Aβ40-WT fibrils labeled with ^13^C-ring Phe19 and U-^13^C Leu34 for the CAA/ad case. The ^13^C-ring Phe19 resonance falls along the diagonal in this region of the 2D NMR spectrum, while the other resonances correspond to cross-peaks between Leu34 and the Phe19 ring.

Figure 6 compares NMR measurements of generation-6 Aβ40-WT fibrils using a 1:1 mixture of Aβ40-WT peptides containing 1-^13^C Val36, 2-^13^C Ala30, and 3-^13^C Ala30 as described above. Upon subsequent rounds of seeded-fibril growth, only the parallel component is observed for generation-6 fibrils in the CAA/ad case (Fig. 6, *A* and *C*), while both parallel, in-register, and antiparallel components are observed for the CAA/s case (Fig. 6*, B* and *D*). In TEM images, the generation-6 fibrils from both cases exhibit a long, twisted morphology (Fig. 6, *E–G*). These fibrils were generally comprised of single twisted protofilaments and twisted pairs of protofilaments. The helical crossing points were generally between 100 and 200 nm. However, a population of highly twisted fibrils with crossing points of less than 50 nm was also observed (Fig. 6*G*). The CAA/s case required one additional round of seeded-fibril growth to generation-7 to yield fibrils with predominantly parallel, in-register structure and a loss of the short, bundled morphology in TEM images (data not shown).

The ability to isolate fibrils that exhibit only the parallel, in-register conformation allows us to probe whether these fibrils are similar to either *in vitro* or brain-derived fibrils. In both Aβ40-WT fibrils grown *in-vitro* (35) and derived from fibril seeds isolated from AD brain, Phe19 often packs against Leu34 (Fig. S5). In brain-derived fibrils, this contact has been observed to be either intramolecular (24) or intermolecular (25). In fibrils exhibiting an intramolecular contact, the hydrophobic C-terminus folds back onto the hydrophobic Leu17-Val18-Phe19-Phe20 sequence in the middle of the Aβ40 peptide. A ^13^C-labeling scheme that probes for this contact involves incorporating ^13^C-ring Phe19 within the LVFF sequence and U-^13^C Leu34 within the hydrophobic C-terminus within the same Aβ40 peptide. Using Aβ40-WT monomer containing these ^13^C-labeled amino acids, generation-6 Aβ40-WT fibrils were derived from generation-5 CAA/ad seeds. The 2D ^13^C NMR spectra (Fig. 6*H*) reveal strong cross-peaks between ^13^C-ring Phe19 and U-^13^C Leu34. To address whether this contact is intramolecular or intermolecular, dilution experiments were performed in which the fibrils were made with 50% ^13^C-labeled peptide and 50% unlabeled peptide. A ∼50% loss of intensity of the ^13^C-ring Phe19 - U-^13^C Leu34 cross-peak relative to the diagonal resonances indicated that the contact is intermolecular (data not shown). As a result, the fibril structure is consistent with intermolecular contacts as observed in the recent cryo-EM structure of Aβ40 fibrils derived from cortical tissue of an AD patient (25). This contact would not be consistent with the cryo-EM structure (36) of fibrils derived from vascular amyloid extracted from meningeal tissue of a patient with severe AD and CAA.

## Discussion

CAA is commonly associated with AD, but also can occur sporadically in the absence of AD. This condition causes vessel damage, neuroinflammation, microhemorrhages, and ischemia, contributing to vascular cognitive impairment and dementia (1, 2). Despite the clinical significance of CAA, little is known about the structure of the fibrils present in amyloid deposits that accumulate on or within cerebral vessels. In this study, we use two different vascular amyloid extraction methods, LCM and blood vessel stripping, for structural analysis. We first demonstrate that the brain-derived fibrils resulting from templated growth arise from fibril seeds and are not products of monomer self-nucleation. We observed a distinctive shift in the amide I vibration assigned to β-sheet secondary structure in FTIR measurements of sporadic CAA amyloid, which is not observed in control vascular tissue lacking amyloid. TEM images of isolated vascular amyloid reveal densely packed networks of short, bundled fibrils with similar morphology. The use of these fibrils as seeds for templated growth produces Aβ40-WT fibrils that have a twisted morphology. Importantly, vascular Aβ40-WT fibrils derived from human CAA cases were found to contain antiparallel β-sheet structure. This observation agrees with the antiparallel structure observed in fibrils derived from vascular amyloid in mice and rats containing the E22Q and D23N Aβ familial mutants (20, 21).

### Laser capture microdissection for isolating brain-derived vascular amyloid

The LCM method for isolating brain-derived vascular amyloid results in highly purified fibrils that can be used as seeds for templated fibril growth. The high purity of the Aβ peptide in these samples facilitates the use of FTIR to distinguish seeded growth from self-nucleation. An advantage of FTIR spectroscopy is that it provides a global, quantitative measure of the conversion of monomer to fibrils. In contrast, the use of TEM is more qualitative for several reasons. First, one may inadvertently observe the original fibrils isolated from the brain tissue. In addition, one cannot distinguish between self-nucleated and seeded fibrils by TEM.

The high concentration of Aβ fibrils in the LCM particles, however, does allow one to use TEM to image the Aβ fibrils within the vascular amyloid particles prior to seeded growth. Negative-stain TEM images of the generation-0 samples show tightly enmeshed fibrils that are relatively short and straight. An important question for structural studies on brain-derived fibrils has been whether templated fibril growth accurately reproduces the original structure of the fibrils within the amyloid deposits. For our studies, we found that the fibrils in generation-1 through generation-3 in the CAA/s case exhibited the morphology of the generation-0 fibrils. However, propagating to generation-6 yielded isolated and twisted fibrils. This morphology was observed for the CAA/ad case already at generation-1. In generations 3 and 6, we also observed a population of highly twisted fibrils having a <50 nm helical twist (see Fig. 6*G*). This unique morphology has been observed in TEM images of fibrils of human-derived Aβ purified directly from the vascular amyloid of three AD patients exhibiting CAA without seeding with monomeric Aβ40-WT (36).

We were not able to dissociate the tightly enmeshed fibrils in the LCM particles with collagenase and proteinase K to yield the long twisted fibrils as in the cryo-EM study (36). The observations that highly twisted fibrils can be observed in the CAA/ad case, the CAA/s case, and in the cryo-EM study suggest that the short, flat generation-0 fibrils adopt this twisted morphology when not interacting with adjacent fibrils or protofibrils. In the case of Aβ42 fibrils that can adopt both twisted fibrils and flat, laterally associated fibrils, electron paramagnetic resonance measurements indicate that the fibrils have the same secondary structure, but the laterally associated fibrils have stronger side chain interactions along the length of the Aβ sequence (37). In the cryo-EM study on fibrils derived from vascular amyloid (36), several polymorphs were observed, which the authors proposed had same internal fold and were distinguished by lateral association.

Nevertheless, there are potentially several alternative explanations as to the appearance of the long, twisted fibrils after seeding through multiple generations. Structural cofactors (*e.g.*, metal ions, lipids, or amyloid-associated proteins) that stabilize specific fibril structures in the context of brain tissue may be diluted upon purification or elongation of the fibrils. Alternatively, the parallel, in-register conformation observed for the generation-6 CAA/ad fibrils and for the generation 7 CAA/s fibrils may be the more stable form and propagate with higher efficiency under our growth conditions. Backbone interactions appear to favor a flat morphology, while side-chain interactions favor a twisted morphology (38). Intermolecular side-chain to side-chain interactions favor the parallel, in-register orientation due to favorable enthalpic interactions when the side chains are aligned. In either case, it is generally recognized that multiple rounds of seeding result in more homogeneous fibril populations (39).

Finally, the LCM method has the advantage that specific vascular amyloid deposits in brain tissue can be excised for seeding and structure analysis. As a result, this approach reduces the potential for contamination from parenchymal amyloid. In future studies, LCM will provide a way to specifically isolate subtypes of both vascular and parenchymal amyloid. Recently, amyloid deposits from specific regions in the brain have been compared and correlated with proteomic analyses using LCM (40).

### Antiparallel and parallel structure in Aβ fibrils in sporadic CAA

We have previously found that vascular amyloid isolated from a CAA transgenic rodent model harboring the E22Q/D23N double mutant of Aβ contains antiparallel β-sheet structure (20, 21). It is known that the D23N mutation enhances the propensity for the Aβ40 peptide to adopt antiparallel β-sheet (18). An open question is whether antiparallel structure is unique to mutants of Aβ40 or is present in CAA deposits comprised of nonmutated, wild-type Aβ40 peptide. Here a comparison has been made on vascular amyloid composed of nonmutated Aβ arising from CAA in patients with and without AD.

Using two different isolation methods on two different brains harboring vascular amyloid deposits, we found that both contain a mixture of antiparallel and parallel, in-register fibrils. The structure of antiparallel Aβ40 fibrils of the Iowa mutant (18) indicates that the fibrils are stabilized by electrostatic interactions between neighboring strands in the fibrils. We have previously suggested that biological membranes provide a way to enhance the electrostatic interactions and stabilize antiparallel fibrils (19). A similar mechanism may be operating in the case of Aβ40-WT fibrils in sporadic CAA. Recently, Aβ40-WT fibrils with mixed antiparallel and parallel structures have been observed in the presence of the ganglioside GM1 (41). Moreover, studies on α-synuclein have indicated that more hydrophobic environments favor antiparallel fibril structure over parallel structure (42).

Importantly, we observe a difference between parenchymal and vascular amyloid with respect to the contribution of the antiparallel β-sheet fibril conformation. This difference is in agreement with antibody (43) and small molecule (44) binding studies and suggests that the structures of fibrillar Aβ in parenchymal and vascular amyloid are different. Besides the Iowa peptide, the Italian variant of the Aβ peptide with a glutamate to lysine substitution at position 22 (E22K) also has a propensity to form antiparallel β-sheet fibrils (45). These studies also proposed a correlation between antiparallel fibril structure and CAA.

We addressed whether the antiparallel fibrils could be seeded to multiple generations. We found that after six generations for the CAA/ad case and seven generations for the CAA/s case, the seeded fibrils had primarily parallel, in-register β-sheet structure. TEM images showed that the fibrils from both cases exhibit a twisted morphology.

Antiparallel β-sheet structure is often associated with Aβ oligomers and protofibrils (30, 46, 47) raising the possibility that we are detecting early intermediates in our experiments, rather than fibrils. Both temperature and concentration dramatically influence the formation of oligomers and protofibrils with higher temperatures (*e.g.*, 37 °C) favoring oligomer/protofibril formation and resulting in fibril formation *via* a nucleated conformational conversion to cross β-sheet structure (48). Lower temperatures favor monomeric Aβ that can polymerize with fibril seeds or self-nucleate to form fibrils *via* a nucleated polymerization mechanism (49). Our studies were carried out at room temperature under quiescent conditions that favor the nucleated polymerization mechanism. The FTIR measurements in Figures 3, S2, and S3 show that under our conditions the Aβ40-WT peptide does not transition to fibrils. Additionally, solid-state NMR experiments were undertaken of hydrated generation-3 fibrils that were separated from nonfibrillar species by centrifugation (Fig. S8). The NMR spectra of these fibrils also exhibited cross-peaks characteristic of antiparallel and parallel, in-register β-sheet structure.

We have not been able to isolate homogeneous antiparallel fibrils of Aβ40-WT through seeded fibril growth under the set of elongation conditions used in this study. One possibility is that the individual fibrils in vascular amyloid contain a mixture of parallel and antiparallel β-sheet conformations. An attribute of fibrils with significant antiparallel character is that they have a flat, nontwisted appearance (19). Flat, nontwisted fibrils with a mixture of parallel and antiparallel β-sheet conformations have also been observed in the presence of the GM1 ganglioside (41). We have shown in recent studies on Aβ40-Iowa, which has a strong propensity to form fibrils composed of antiparallel β-sheet structure, that strong agitation and temperature drive the conversion of antiparallel β-structure to parallel structure (19, 50). The room temperature, quiescent conditions used here favor antiparallel structure over parallel, in-register structure. In a similar fashion, a low ionic strength buffer favors antiparallel β-sheet structure in the Aβ40-Iowa peptide, although the effect is less pronounced than agitation and temperature (unpublished results).

The generation-0 fibrils observed in the LCM samples are short, nontwisted fibrils with a high degree of lateral association. The antiparallel contribution to the fibrils may be the origin of their flat morphology as a result of stronger backbone hydrogen bonding interactions (38). The antiparallel contribution appears to be lost as the fibrils are seeded to multiple generations. Cofactors (*e.g.*, lipids, metal ions) present in vascular amyloid that may stabilize antiparallel structure may be diluted by seeding through multiple generations. Another possibility is that the parallel, in-register structure has higher stability and faster elongation rates, perhaps due to an increase of interactions along the length of the fibril (51). The antiparallel structure found in Aβ40-Iowa is only stabilized by interactions within the β-sheet core of the fibril (residues 15–40) and does not appear to have contributions from the N-terminus. Nanoscale FTIR-AFM experiments are in progress that will allow us to establish if individual fibrils contain regions of parallel and antiparallel structure (52).

Regardless of the origin, the presence of coexisting antiparallel and parallel, in-register β-sheet structure indicates another level of polymorphism that can exist in brain-derived amyloid fibrils. Understanding the clearance mechanism of the Aβ peptides *via* the cerebral vasculature and how different brain cofactors contribute to fibril structure will be important areas of investigation toward understanding the differences between vascular and parenchymal amyloid.

## Experimental procedures

### Peptide synthesis

Aβ peptides were synthesized using tBOC-chemistry ERI-Amyloid and purified by high-performance liquid chromatography using linear water−acetonitrile gradients containing 0.1% (v/v) trifluoroacetic acid. The mass of the purified peptide was measured using matrix-assisted laser desorption or electrospray ionization mass spectrometry and was consistent with the calculated mass for the peptide. On the basis of analytical reverse-phase high-performance liquid chromatography and mass spectrometry, the purity of the peptides was 95–99%.

### Sample preparation

Monomeric Aβ peptide was prepared by first dissolving purified peptides in 1,1,1,3,3,3-hexafluoro-2-propanol (HFIP), flash freezing in liquid nitrogen, and then lyophilizing under a 25 mTorr vacuum for at least 2 days. Lyophilized Aβ peptides were dissolved in a small volume of 50 mM NaOH for 1 h at 25 °C under quiescent conditions, diluted in seeding buffer (10 mM sodium phosphate, 0 mM NaCl, pH 7.4) at 4 °C to the desired final Aβ concentration, and then filtered with 0.2 μm filters immediately prior to use. The extracted human tissue samples were first suspended in seeding buffer to a final volume of 500 μl and then sonicated for 4 s every 3 min for a duration of 22 min at 30% power on ice with a 1/8″ diameter probe tip (Fisher Scientific, Model 50).

### Aβ temperature and concentration

Monomeric Aβ40-WT was dissolved to a final concentration of 100 μM as described above. Aβ40-WT peptide monomers were incubated in the presence of human-derived material at 25 °C and were subject to intermittent sonication for 10 s every hour for 24 h at <10% power. Samples were then incubated at 25 °C under quiescent conditions for 4–7 days postsonication for the development of generation-1 fibrils. Subsequent generations were developed by incubating fresh Aβ40-WT peptide monomers with either 5% or 15% (w/w) of parental Aβ40-WT fibrils at 25 °C under quiescent conditions for 1–2 weeks.

### Fourier-transformed infrared spectroscopy

FTIR measurements were made using a Bruker Vertex 70v spectrometer with an attenuated total reflectance (ATR) accessory and room temperature detector. Samples were layered on a 4 mm germanium ATR plate (Pike Technologies) using 50 μl of the peptide solution (∼25 μg) and then dried with compressed air. The spectral resolution was 4 cm^−1^ and typically 500 scans were averaged per spectrum. For data presentation, FTIR spectra were normalized to the intensity of the amide II absorbance band (∼1530–1550 cm^−1^).

### Thioflavin-T fluorescence spectroscopy

Fluorescence measurements were taken using a Spectra Max iD3 spectrometer. Peptide solutions corresponding to 100 μM total Aβ were used for kinetic fluorescent studies. A final concentration of 37.5 μM thioflavin-T was used with an excitation wavelength of 440 nm and an emission wavelength of 490 nm. A total of 200 μl of the Aβ and thioflavin-T solutions was added to each corresponding well in a 96-well clear (Greiner) microplate. Measurements were taken from the bottom of the plate every 10 min for 24–48 h with 2 s low orbital shaking in-between reads. The OD setting was set to 1.

### Solid-state NMR spectroscopy

Room-temperature solid-state MAS NMR experiments were performed at a ^13^C frequency of 125 MHz on a Bruker AVANCE spectrometer using 4 mm MAS probes. The MAS spinning rate was set to 10 KHz (±5 Hz). Ramped amplitude cross-polarization was used with a contact time of 2 ms. The ^13^C field strength was 54.4 kHz and ramped ^1^H field was centered at approximately 50 kHz. Two-pulse phase-modulated decoupling was used during the evolution and acquisition periods with a radio-frequency field strength of 82.7 kHz. Internuclear ^13^C–^13^C distance constraints were obtained from 2D DARR NMR experiments (53) using a mixing time of 600 ms. Each data set contained 64 t_1_ increments and 1024 complex t_2_ points with spectral widths of 27.7 kHz in both dimensions. In total, 512 scans were averaged per t_1_ increment.

All ^13^C solid-state MAS NMR spectra were externally referenced to the ^13^C resonance of neat TMS at 0 ppm at room temperature. Using TMS as the external reference, we calibrated the carbonyl resonance of solid glycine at 176.46 ppm. The chemical shift difference of ^13^C of DSS in D_2_O relative to neat TMS is 2.01 ppm.

### Transmission electron microscopy

Samples were diluted, deposited onto carbon-coated copper mesh grids, and negatively stained with 2% (w/v) uranyl formate. The excess stain was blotted away, and the sample grids were allowed to air dry. The samples were viewed with an FEI Tecnai 12 BioTwin 80 kV transmission electron microscope, and digital images were taken with an Advanced Microscopy Techniques camera.

### Biochemical isolation of cerebral vascular amyloid deposits from postmortem brain tissue

The brain tissues used in this study were provided by the University of California Irvine Alzheimer's Disease Research Center (UCI-ADRC) and the Institute for Memory Impairments and Neurological Disorders. Tissues used in these studies were from: a 76-year-old male AD patient; a 71-year-old male sporadic CAA patient; and a 70-year-old male control patient. Brain vessels were isolated from ≈1 g of brain cortices. The brain tissue was homogenized in sterile Tris-HCl buffered saline (TBS) using a Kontes 7 ml glass Dounce homogenizer. The brain tissue homogenate was centrifuged at 16,000*g* for 5 min, and the aqueous layer was removed and discarded. The pellet was washed with sterile TBS and rehomogenized in a solution of sterile 17% Dextran/TBS (MW 65,000–85,000). The homogenate was centrifuged at 10,000*g* for 10 min. The lipid top layer and supernatant were discarded, and the vessel pellet was washed with sterile TBS with centrifugation at 16,000*g* for 5 min. The washed vessel pellet was resuspended in sterile TBS and passed through a 20 μm Pluristrainer filter (PluriSelect) to collect the vessels. The filter was removed from the holder, and the vessels were washed off the filter using sterile TBS and collected in a 1.5 ml microfuge tube. The vessel sample was centrifuged at 16,000*g* for 5 min, and the liquid was discarded. The concentrated vessel pellet was resuspended in 100 μl of TBS.

To confirm the presence of amyloid-containing vessels, a droplet of the vessel sample was dried on a glass slide and immunostained with goat anti-collagen IV (1:500; Novus) and 0.015% thioflavin S overnight. After washing with PBS, the sample was incubated for 2 h in the dark with AexaFluor594-labeled donkey anti-goat secondary antibody (1:1000; Invitrogen) and then imaged with BZ-X600 Fluorescent Microscope (Keyence).

To isolate fibrillar amyloid deposits, the vessel samples were digested at 37 °C overnight with 6 mg/ml collagenase type 2 (Worthington Biochem. Corp., Lakewood, NJ), 100 μg/ml Roche DNase I, and Proteinase K (1:1000; IBI Sci). The digested sample was centrifuged at 16,000*g* for 5 min, and the resulting pellet was washed with sterile TBS and resuspended in 100 μl of TBS. To confirm elimination of vessel material from the amyloid deposit, a droplet of the pellet sample was dried on a glass slide and immunostained for collagen and thioflavin S as described above.

### Laser-capture microdissection (LCM) of cerebral vascular amyloid deposits from human brain

Fresh frozen postmortem brain tissues were sectioned at 25 μm and mounted on Leica Frame Slides (Leica Microsystems). Mounted brain sections were stained with thioflavin S to identify cerebral vascular fibrillar amyloid. For each case, a total of 700 individual amyloid-containing cerebral vessels were identified, excised, and captured using a LMD6 laser capture microdissection microscope LMD6 (Leica Microsystems). The dissected vascular amyloid deposits were collected into 100 μl of 10 mM sodium phosphate buffer, pH 7.2, containing 0.01% sodium azide.

### Aβ ELISA of isolated cerebral vascular amyloid

For the analysis of Aβ composition, 10 μl of the collected vascular amyloid suspension was added to 90 μl 5 M guanidine HCl, 50 mM Tris-HCl, pH 8.0. The samples were then diluted 1:200 and subjected to sandwich ELISA for the measurement of Aβ40 and Aβ42 peptides as described (54, 55). Briefly, in the sandwich ELISAs Aβ40 and Aβ42 were captured using their respective carboxyl-terminal specific antibodies mAb2G3 and mAb21F12 and biotinylated m3D6, specific for N-terminus of human Aβ, was used for detection (54). Each vascular amyloid sample was measured in triplicate and compared with linear standard curves generated with known concentrations of human Aβ40 and Av42 using a Spectramax M2 plate reader (Molecular Devices).

## Data availability

All data contained within the manuscript, or referred to as unpublished, are available upon request. Contact: Steven Smith (steven.o.smith@stonybrook.edu).

## Supporting information

This article contains supporting information (23, 56).

## Conflict of interest

The authors declare that they have no conflicts of interest with the contents of this article.

## References

[1] Thal D.R., Ghebremedhin E., Rub U., Yamaguchi H., Del Tredici K., Braak H. (2002). Two types of sporadic cerebral amyloid angiopathy. J. Neuropath. Exper. Neuro..

[2] Greenberg S.M., Bacskai B.J., Hernandez-Guillamon M., Pruzin J., Sperling R., van Veluw S.J. (2020). Cerebral amyloid angiopathy and Alzheimer's disease - one peptide, two pathways. Nat. Rev. Neuro..

[3] Greenberg S.M., Salman R.A.S., Biessels G.J., van Buchem M., Cordonnier C., Lee J.M., Montaner J., Schneider J.A., Smith E.E., Vernooij M., Werring D.J. (2014). Outcome markers for clinical trials in cerebral amyloid angiopathy. Lancet Neurol..

[4] Brenowitz W.D., Nelson P.T., Besser L.M., Heller K.B., Kukull W.A. (2015). Cerebral amyloid angiopathy and its co-occurrence with Alzheimer's disease and other cerebrovascular neuropathologic changes. Neurobiol. Aging.

[5] Murphy M.P., LeVine H. (2010). Alzheimer's disease and the amyloid-β peptide. J. Alzheimers Dis..

[6] Xu X.M. (2009). Gamma-secretase catalyzes sequential cleavages of the AβPP transmembrane domain. J. Alzheimers Dis..

[7] Walsh D.M., Klyubin I., Fadeeva J.V., Cullen W.K., Anwyl R., Wolfe M.S., Rowan M.J., Selkoe D.J. (2002). Naturally secreted oligomers of amyloid β protein potently inhibit hippocampal long-term potentiation *in vivo*. Nature.

[8] Miller D.L., Papayannopoulos I.A., Styles J., Bobin S.A., Lin Y.Y., Biemann K., Iqbal K. (1993). Peptide compositions of the cerebrovascular and senile plaque core amyloid deposits of Alzheimer's disease. Arch. Biochem. Biophys..

[9] Makela M., Paetau A., Polvikoski T., Myllykangas L., Tanskanen M. (2016). Capillary amyloid-β protein deposition in a population-based study (Vantaa 85+). J. Alzheimers Dis..

[10] Wattendorff A.R., Frangione B., Luyendijk W., Bots G.T.A.M. (1995). Hereditary cerebral haemorrhage with amyloidosis, Dutch type (HCHWA-D): Clinicopathological studies. J. Neuro. Neurosurg. Psych..

[11] Bornebroek M., Van Buchem M.A., Haan J., Brand R., Lanser J.B., de Bruine F.T., Roos R.A. (1996). Hereditary cerebral hemorrhage with amyloidosis-Dutch type: Better correlation of cognitive deterioration with advancing age than with number of focal lesions or white matter hyperintensities. Alzheimers Dis. Associated Disord..

[12] Levy E., Carman M.D., Fernandez-Madrid I.J., Power M.D., Lieberburg I., van Duinen S.G., Bots G.T., Luyendijk W., Frangione B. (1990). Mutation of the Alzheimer's disease amyloid gene in hereditary cerebral hemorrhage, Dutch type. Science.

[13] Van Broeckhoven C., Haan J., Bakker E., Hardy J.A., Van Hul W., Wehnert A., Vegter-Van der Vlis M., Roos R.A. (1990). Amyloid β protein precursor gene and hereditary cerebral hemorrhage with amyloidosis (Dutch). Science.

[14] van Duinen S.G., Castano E.M., Prelli F., Bots G.T., Luyendijk W., Frangione B. (1987). Hereditary cerebral hemorrhage with amyloidosis in patients of Dutch origin is related to Alzheimer disease. Proc. Natl. Acad. Sci. U. S. A..

[15] Maat-Schieman M.L., van Duinen S.G., Rozemuller A.J., Haan J., Roos R.A. (1997). Association of vascular amyloid β and cells of the mononuclear phagocyte system in hereditary cerebral hemorrhage with amyloidosis (Dutch) and Alzheimer disease. J. Neuropathol. Exp. Neuro..

[16] Maat-Schieman M.L., Yamaguchi H., Hegeman-Kleinn I.M., Welling-Graafland C., Natté R., Roos R.A., van Duinen S.G. (2004). Glial reactions and the clearance of amyloid β protein in the brains of patients with hereditary cerebral hemorrhage with amyloidosis-Dutch type. Acta Neuropathol..

[17] Rozemuller A.J., Roos R.A., Bots G.T., Kamphorst W., Eikelenboom P., Van Nostrand W.E. (1993). Distribution of β/A4 protein and amyloid precursor protein in hereditary cerebral hemorrhage with amyloidosis-Dutch type and Alzheimer's disease. Amer. J. Path..

[18] Qiang W., Yau W.-M., Luo Y., Mattson M.P., Tycko R. (2012). Antiparallel β-sheet architecture in Iowa-mutant β-amyloid fibrils. Proc. Natl. Acad. Sci. USA.

[19] Crooks E.J., Irizarry B.A., Ziliox M., Kawakami T., Victor T., Xu F., Hojo H., Chiu K., Simmerling C., Van Nostrand W.E., Smith S.O., Miller L.M. (2020). Copper stabilizes antiparallel β-sheet fibrils of the amyloid-β40 (Aβ40)-Iowa variant. J. Biol. Chem..

[20] Xu F., Fu Z., Dass S., Kotarba A.E., Davis J., Smith S.O., Van Nostrand W.E. (2016). Cerebral vascular amyloid seeds drive amyloid β-protein fibril assembly with a distinct anti-parallel structure. Nat. Comm.

[21] Davis J., Xu F., Hatfield J., Lee H., Hoos M.D., Popescu D., Crooks E., Kim R., Smith S.O., Robinson J.K., Benveniste H., Van Nostrand W.E. (2018). A novel transgenic rat model of robust cerebral microvascular amyloid with prominent vasculopathy. Amer. J. Path..

[22] Boon B.D.C., Bulk M., Jonker A.J., Morrema T.H.J., van den Berg E., Popovic M., Walter J., Kumar S., van der Lee S.J., Holstege H., Zhu X.Y., Van Nostrand W.E., Natte R., van der Weerd L., Bouwman F.H. (2020). The coarse-grained plaque: A divergent Aβ plaque-type in early-onset Alzheimer's disease. Acta Neuropathol..

[23] Lu J.-X., Qiang W., Yau W.-M., Schwieters C.D., Meredith S.C., Tycko R. (2013). Molecular structure of beta-amyloid fibrils in Alzheimer's disease brain tissue. Cell.

[24] Paravastu A.K., Qahwash I., Leapman R.D., Meredith S.C., Tycko R. (2009). Seeded growth of β-amyloid fibrils from Alzheimer's brain-derived fibrils produces a distinct fibril structure. Proc. Natl. Acad. Sci. U. S. A..

[25] Ghosh U., Thurber K.R., Yau W.M., Tycko R. (2021). Molecular structure of a prevalent amyloid-β fibril polymorph from Alzheimer's disease brain tissue. Proc. Natl. Acad. Sci. U. S. A..

[26] Petkova A.T., Leapman R.D., Guo Z.H., Yau W.M., Mattson M.P., Tycko R. (2005). Self-propagating, molecular-level polymorphism in Alzheimer's β-amyloid fibrils. Science.

[27] Bitan G., Kirkitadze M.D., Lomakin A., Vollers S.S., Benedek G.B., Teplow D.B. (2003). Amyloid β-protein (Aβ) assembly: Aβ40 and Aβ42 oligomerize through distinct pathways. Proc. Natl. Acad. Sci. U. S. A..

[28] Miyazawa T., Blout E.R. (1961). The infrared spectra of polypeptides in various conformations: amide I and amide II bands. J. Am. Chem. Soc..

[29] Jackson M., Mantsch H.H. (1995). The use and misuse of FTIR spectroscopy in the determination of protein structure. Crit. Rev. Biochem. Mol. Biol..

[30] Sarroukh R., Cerf E., Derclaye S., Dufrene Y.F., Goormaghtigh E., Ruysschaert J.M., Raussens V. (2011). Transformation of amyloid β(1-40) oligomers into fibrils is characterized by a major change in secondary structure. Cell. Mol. Life Sci..

[31] Baldus M. (2002). Correlation experiments for assignment and structure elucidation of immobilized polypeptides under magic angle spinning. Prog. NMR Spec..

[32] Ghosh U., Yau W.M., Tycko R. (2018). Coexisting order and disorder within a common 40-residue amyloid-β fibril structure in Alzheimer's disease brain tissue. Chem. Commun..

[33] Tycko R., Sciarretta K.L., Orgel J.P.R.O., Meredith S.C. (2009). Evidence for novel β-sheet structures in Iowa-mutant β-amyloid fibrils. Biochemistry.

[34] Sgourakis N.G., Yau W.M., Qiang W. (2015). Modeling an in-register, parallel “Iowa” Aβ fibril structure using solid-state NMR data from labeled samples with rosetta. Structure.

[35] Petkova A.T., Ishii Y., Balbach J.J., Antzutkin O.N., Leapman R.D., Delaglio F., Tycko R. (2002). A structural model for Alzheimer's β-amyloid fibrils based on experimental constraints from solid state NMR. Proc. Natl. Acad. Sci. U. S. A..

[36] Kollmer M., Close W., Funk L., Rasmussen J., Bsoul A., Schierhorn A., Schmidt M., Sigurdson C.J., Jucker M., Fandrich M. (2019). Cryo-EM structure and polymorphism of Aβ amyloid fibrils purified from Alzheimer's brain tissue. Nat. Comm..

[37] Wang H.S., Duo L., Hsu F., Xue C., Lee Y.K., Guo Z.F. (2020). Polymorphic Aβ42 fibrils adopt similar secondary structure but differ in cross-strand side chain stacking interactions within the same β-sheet. Sci. Rep..

[38] Periole X., Huber T., Bonito-Oliva A., Aberg K.C., van der Wel P.C.A., Sakmar T.P., Marrink S.J. (2018). Energetics underlying twist polymorphisms in amyloid fibrils. J. Phys. Chem. B.

[39] Paravastu A.K., Leapman R.D., Yau W.M., Tycko R. (2008). Molecular structural basis for polymorphism in Alzheimer's β-amyloid fibrils. Proc. Natl. Acad. Sci. U. S. A..

[40] Hondius D.C., Eigenhuis K.N., Morrema T.H.J., van der Schors R.C., van Nierop P., Bugiani M., Li K.W., Hoozemans J.J.M., Smit A.B., Rozemuller A.J.M. (2018). Proteomics analysis identifies new markers associated with capillary cerebral amyloid angiopathy in Alzheimer's disease. Acta Neuro. Comm..

[41] Okada Y., Okubo K., Ikeda K., Yano Y., Hoshino M., Hayashi Y., Kiso Y., Itoh-Watanabe H., Naito A., Matsuzaki K. (2019). Toxic amyloid tape: A novel mixed antiparallel/parallel β-aheet structure formed by amyloid β-protein on GM1 clusters. ACS Chem. Neurosci..

[42] Camino J.D., Gracia P., Chen S.W., Sot J., de la Arada I., Sebastian V., Arrondo J.L.R., Goni F.M., Dobson C.M., Cremades N. (2020). The extent of protein hydration dictates the preference for heterogeneous or homogeneous nucleation generating either parallel or antiparallel β-sheet alpha-synuclein aggregates. Chem. Sci..

[43] Rutgers K.S., van Remoortere A., van Buchem M.A., Verrips C.T., Greenberg S.M., Bacskai B.J., Frosch M.P., van Duinen S.G., Maat-Schieman M.L., van der Maarel S.M. (2011). Differential recognition of vascular and parenchymal β amyloid deposition. Neuro. Aging.

[44] Han B.H., Zhou M.L., Vellimana A.K., Milner E., Kim D.H., Greenberg J.K., Chu W.H., Mach R.H., Zipfel G.J. (2011). Resorufin analogs preferentially bind cerebrovascular amyloid: Potential use as imaging ligands for cerebral amyloid angiopathy. Mol. Neurodegen..

[45] Miravalle L., Tokuda T., Chiarle R., Giaccone G., Bugiani O., Tagliavini F., Frangione B., Ghiso J. (2000). Substitutions at codon 22 of Alzheimer's Aβ peptide induce diverse conformational changes and apoptotic effects in human cerebral endothelial cells. J. Biol. Chem..

[46] Cerf E., Sarroukh R., Tamamizu-Kato S., Breydo L., Derclaye S., Dufrene Y.F., Narayanaswami V., Goormaghtigh E., Ruysschaert J.M., Raussens V. (2009). Antiparallel β-sheet: A signature structure of the oligomeric amyloid β-peptide. Biochem. J..

[47] Huang D.T., Zimmerman M.I., Martin P.K., Nix A.J., Rosenberry T.L., Paravastu A.K. (2015). Antiparallel β-sheet structure within the C-terminal region of 42-residue Alzheimer's amyloid-β peptides when they form 150-kDa oligomers. J. Mol. Biol..

[48] Fu Z., Aucoin D., Davis J., Van Nostrand W.E., Smith S.O. (2015). Mechanism of nucleated conformational conversion of Aβ42. Biochemistry.

[49] Fawzi N.L., Ying J.F., Torchia D.A., Clore G.M. (2010). Kinetics of amyloid β monomer-to-oligomer exchange by NMR relaxation. J. Am. Chem. Soc..

[50] Fu Z., Van Nostrand W.E., Smith S.O. (2021). Anti-parallel β-hairpin structure in soluble Aβ oigomers of Aβ40-Dutch and Aβ40-Iowa. Int. J. Mol. Sci..

[51] Kodali R., Williams A.D., Chemuru S., Wetzel R. (2010). Aβ(1-40) forms five distinct amyloid structures whose β-sheet contents and fibril stabilities are correlated. J. Mol. Biol..

[52] Waeytens J., Van Hemelryck V., Deniset-Besseau A., Ruysschaert J.M., Dazzi A., Raussens V. (2020). Characterization by nano-infrared spectroscopy of individual aggregated species of amyloid proteins. Molecules.

[53] Takegoshi K., Nakamura S., Terao T. (2001). ^13^C-^1^H dipolar-assisted rotational resonance in magic-angle spinning NMR. Chem. Phys. Lett..

[54] Johnson-Wood K., Lee M., Motter R., Hu K., Gordon G., Barbour R., Khan K., Gordon M., Tan H., Games D., Lieberburg I., Schenk D., Seubert P., McConlogue L. (1997). Amyloid precursor protein processing and Aβ(42) deposition in a transgenic mouse model of Alzheimer disease. Proc. Natl. Acad. Sci. U. S. A..

[55] DeMattos R.B., O'Dell M A., Parsadanian M., Taylor J.W., Harmony J.A., Bales K.R., Paul S.M., Aronow B.J., Holtzman D.M. (2002). Clusterin promotes amyloid plaque formation and is critical for neuritic toxicity in a mouse model of Alzheimer's disease. Proc. Natl. Acad. Sci. U. S. A..

[56] Qiang W., Yau W.M., Lu J.X., Collinge J., Tycko R. (2017). Structural variation in amyloid-β fibrils from Alzheimer's disease clinical subtypes. Nature.

