# Supplementary material for: Human cerebral vascular amyloid contains both antiparallel and parallel in-register Aβ40 fibrils
Source: J Biol Chem. 2021 Sep 29;297(5):101259. doi: 10.1016/j.jbc.2021.101259 (PMC8528725; doi:10.1016/j.jbc.2021.101259)
Supplement: Supplementary file 1 — Figures S1–S8 [file mmc1.pdf]

Supporting Information

Human cerebral vascular amyloid contains both anti-parallel and parallel, in-register Aβ40 fibrils

Brandon A. Irizarry[‡], Judianne Davis[§], Xiaoyue Zhu[§], Baayla D.C. Boon[†,¶], Annemieke J.M. Rozemuller[¶], William E. Van Nostrand[§] and Steven O. Smith[‡,1]

Figure S1. Parallel, in-register and anti-parallel fibrils observed using fibril seeds isolated from the CAA/ad and CAA/s cases by vessel stripping.

Figure S2. Intermittent sonication enhances templated fibril growth.

Figure S3. Harsh sonication nucleates Aβ40-WT fibril growth.

Figure S4. Morphological analysis of vascular-derived Aβ40-WT fibrils.

Figure S5. Molecular structures of Aβ fibrils illustrating the relative positions of Phe19 and Leu34.

Figure S6. Structural propogation of Aβ40-WT fibrils.

Figure S7. Parallel, in-register fibrils seeded from parenchymal amyloid obtained from an atypical AD patient.

Figure S8. Presence of anti-parallel structure in fully hydrated fibrils.

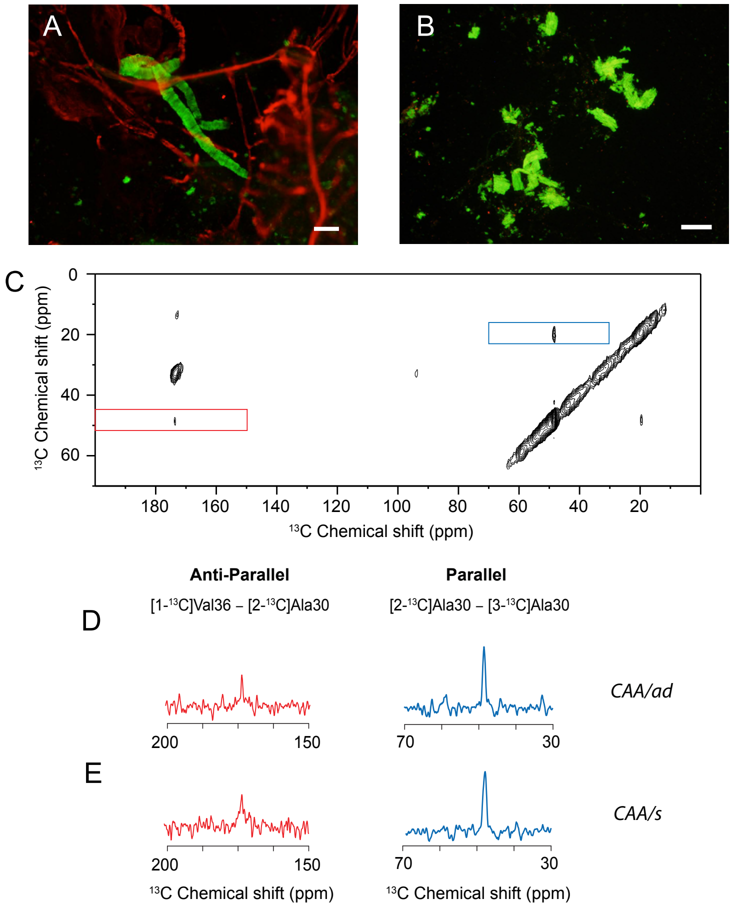

Figure S1. Parallel, in-register and anti-parallel fibrils observed using fibril seeds isolated from the CAA/ad and CAA/s cases by vessel stripping. *A,* Cerebral blood vessels were isolated by mechanical stripping from brain tissue (see Experimental Procedures). The isolated vessels were stained with thioflavin-S to identify vascular amyloid (green) and immunolabeled with an antibody to collagen IV to identify cerebral blood vessels (red). Scale bar = 100 μm. *B,* The isolated blood vessels were treated with collagenase to free the vascular amyloid. Thioflavin-S staining allowed the isolated vascular amyloid to be visualized after sequential purification and collagenase-treatment. Immunolabeling the sample for collagen IV demonstrated the removal of vascular tissue. Scale bar = 100 μm. *C*, Region of the 2D NMR spectrum of generation-3 Aβ40-WT fibrils derived from the CAA/ad case using stripped-vessel isolation. Cross peaks corresponding to fibrils having anti-parallel β-sheet structure and parallel, in-register β-sheet structure are located within the red and blue boxes, respectively. *D-E,* Rows are shown through the diagonal resonances of 2-^{13}C Ala30 (48.6 ppm) and 3-^{13}C Ala30 (20.1 ppm) of generation-3 Aβ40-WT fibrils derived from the CAA/ad (*D*) and CAA/s (*E*) cases.

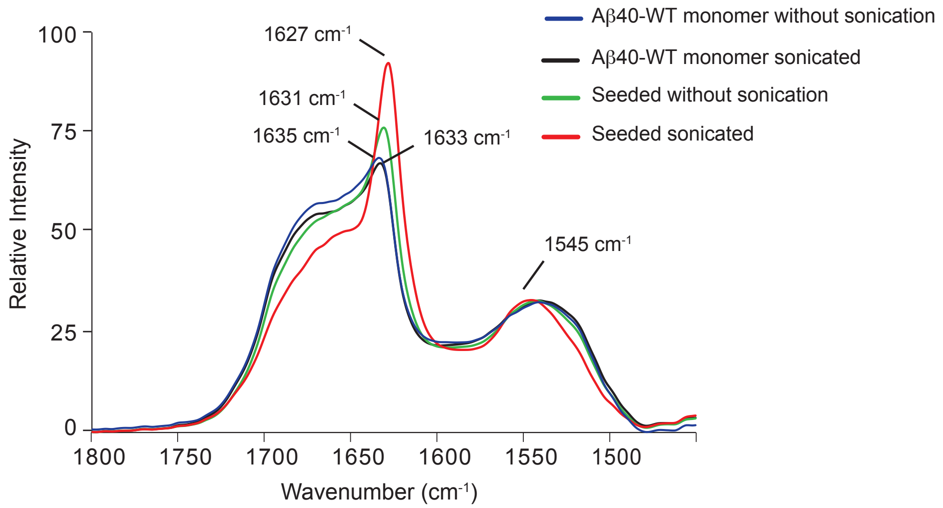

Figure S2. Intermittent sonication enhances templated fibril growth. Intermittent sonication fragments the templated fibrils to generate more nucleation points for Aβ monomers to be incorporated into fibrils during elongation (23,56). To illustrate the effect of intermittent sonication on templated fibril growth, we prepared solutions containing Aβ40-WT monomer in the presence or absence of less than 1% (w/w) pre-formed Aβ40-WT fibrils (i.e. seeds) that were either not sonicated or subject to low power intermittent sonication for 24 h (see Experimental Procedures). Otherwise, the samples were incubated under quiescent conditions at 25 ˚C. FTIR spectra are shown after incubation for 4 d under these four conditions.

The FTIR spectra of sonicated (black trace) and non-sonicated Aβ40-WT monomer (blue trace) samples both exhibit a broad band between 1640 and 1690 cm^{-1}, which is attributed to random coil structure. These spectra indicate that the Aβ40-WT monomers do not transition to mature fibrils under these incubation conditions. In contrast, the spectrum of the sonicated Aβ40-WT seeded sample is markedly different (red trace). This spectrum contains an amide I band characteristic β-sheet at 1627 cm^{-1}. The shift of the amide II band from ~1535 cm^{-1} to 1545 cm^{-1} reflects the conversion of monomeric or oligomeric Aβ to fibrils. Without intermittent sonication, the FTIR spectrum of the seeded Aβ40-WT sample (green trace) exhibits an amide I band at 1631 cm^{-1} with intensity falling between the other two samples. Further, this sample lacks a shift in the amide II region. These results highlight the use of intermittent sonication for enhancing the rate of seeded growth in Aβ peptide samples that contain <5% (w/w) of parental fibrils. Spectra were normalized to the intensity of the amide II absorbance band.

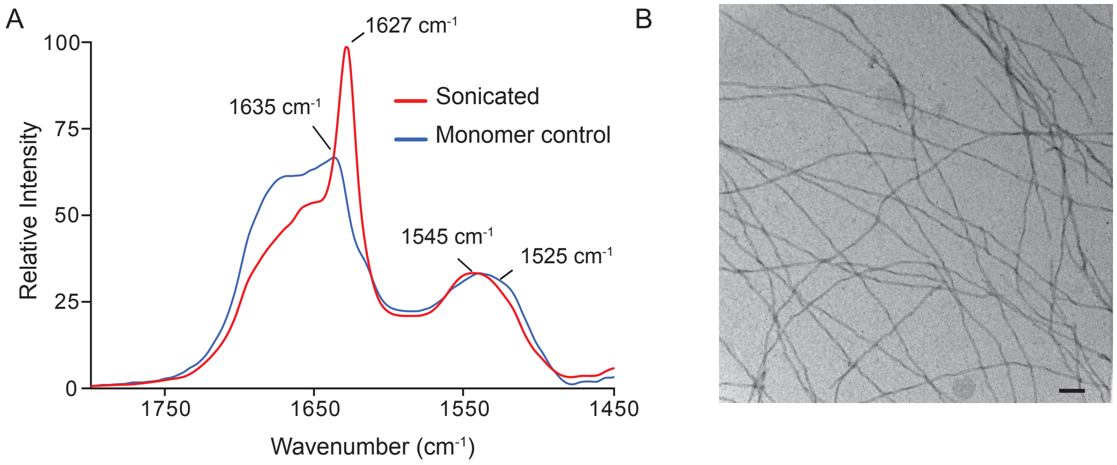

Figure S3. Harsh sonication nucleates Aβ40-WT fibril growth. *A*, To illustrate the potential for sonication to nucleate fibril growth of Aβ40-WT monomer, FTIR spectra are compared of Aβ40-WT monomer subject to strong sonication or left under quiescent conditions without sonication. Specifically, Aβ40-WT monomer (100 μM) was incubated at 25 ˚C for 4 h under quiescent conditions and then split into two samples. One Aβ40-WT sample was subject to sonication for a total of 90 s at 30% power, while the second sample was not sonicated. Both samples were then allowed to incubate at 25 ˚C under quiescent conditions for an additional 20 h. After a total of 24 h, the spectrum of the sonicated Aβ40-WT sample exhibits a characteristic amide I β-sheet band at 1627 cm^{-1} and amide II band at 1545 cm^{-1}, indicating the formation of mature fibrils (red trace). In contrast, the FTIR spectrum corresponding to the non-sonicated Aβ40-WT sample after 24 h exhibits a broad band between 1640 and 1690 cm^{-1}, which is attributed to random coil structure (blue trace). Spectra were normalized to the intensity of the amide II absorbance band. *B,* The sonicated Aβ40-WT sample exhibits long homogenous twisted fibrils after 24 h of incubation by negative-stain TEM. Scale bar = 100 nm. These results illustrate the need to calibrate sonication power levels and duration to reduce the self-nucleation of Aβ40-WT monomers.

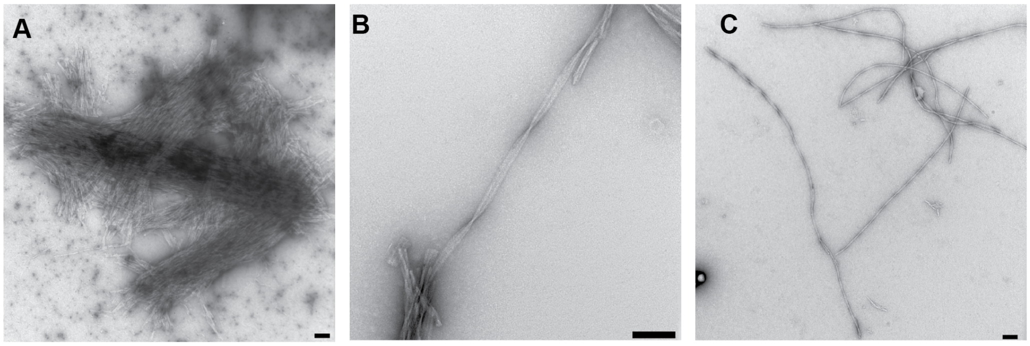

Table 1	CAA/ad		CAA/s	
	Bundled	Twisted	Bundled	Twisted
Generation-1	6	28	29	1
	4	30	30	3
Generation-3	0	30	30	14
	0	30	28	11
Generation-6	0	30	3	30
	0	30	2	29

Figure S4. Morphological analysis of vascular-derived Aβ40-WT fibrils. *A-C,* Negative stain TEM images of vascular-derived Aβ40-WT fibrils from the CAA/ad and CAA/s cases that illustrate the different fibril morphologies observed in generations 1-6. Scale bars correspond to 100 nm. We quantified the morphological distribution of Aβ40-WT fibrils at different fibril generations by separating fibril morphologies into two categories, either short, bundled fibrils (*A)* or long, twisted fibrils (*B*,*C).* Thirty grid squares in several different regions of the stained grids were analyzed to obtain a representative sampling of the fibril populations. Each grid was screened for the presence of either bundled or twisted fibrils. The presence of either morphology within a single grid square was recorded once regardless of the *number* of fibrils with that specific morphology. If both morphologies were present within a single grid square they both were scored. Two independent samples were analyzed for generations 1, 3 and 6 of both the CAA/ad and CAA/s cases. In general, the population of short, bundled fibrils decreased with successive generations, while the population of long, twisted fibrils increased. The distributions, however, were very different across generations for the two CAA cases.

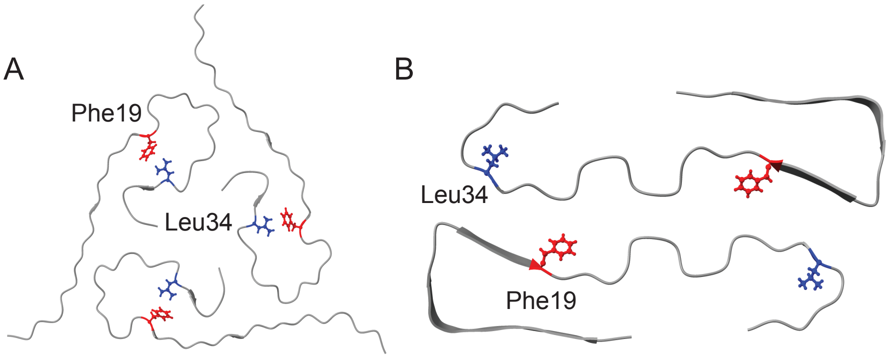

Figure S5. Molecular structures of Aβ fibrils illustrating the relative positions of Phe19 and Leu34. Phe19 and Leu34 often have intra-molecular and inter-molecular packing interactions in Aβ40-WT fibrils. *A,* Human-derived Aβ40-WT fibril propagated from brain parenchymal amyloid of an AD patient (PDB ID 2M4J) illustrate intra-molecular packing of Phe19 and Leu34. This packing geometry is also often observed in Aβ40-WT fibrils formed *in vitro*. *B,* Human-derived Aβ40-WT fibril purified from vascular amyloid obtained from meningeal tissue of an AD patient (PDB ID 6SHS). In comparison to (*A*), neither intra-molecular nor inter-molecular contacts between Phe19 and Leu34 are present. In these fibrils, the individual peptides adopt a more extended conformation.

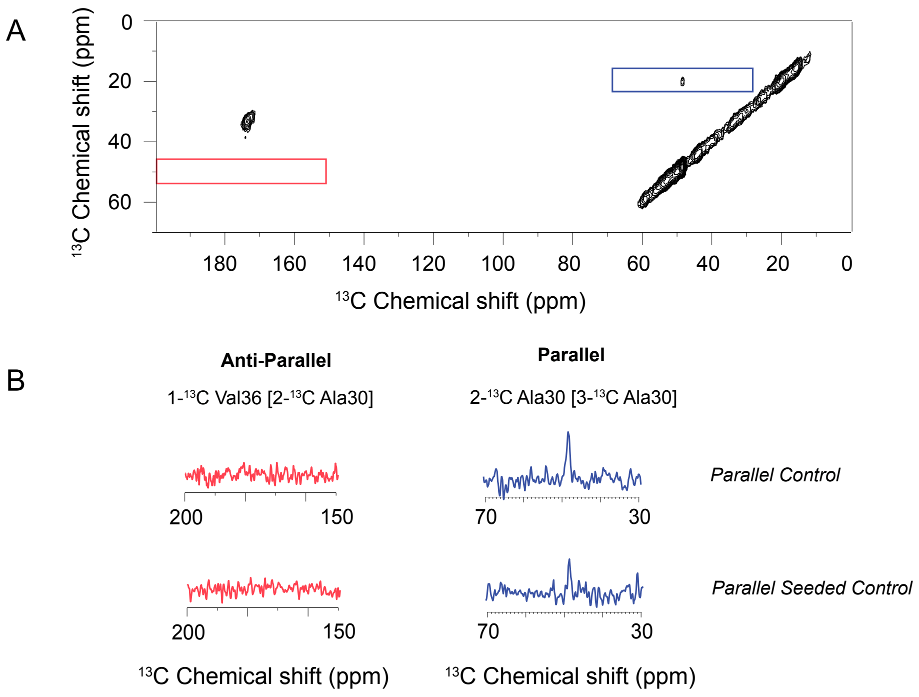

Figure S6. Structural propogation of Aβ40-WT fibrils. *A*, Region of the 2D NMR spectrum of parallel Aβ40-WT parental fibrils. *B,* Rows are shown through the diagonal resonances as in panel (*A*) corresponding to Aβ40-WT parental (parallel control) and daughter (parallel seeded control) fibrils. The location of the rows shown correspond approximately to the positions of the colored boxes in the 2D NMR spectrum. *In vitro* parental Aβ40-WT fibrils were first developed for these experiments through multiple rounds of templated fibril growth and screened for parallel, in register fibril structure (Panel *B*, parallel control). Next, a small amount of the parental Aβ40-WT fibril seeds (~1 μg) was added to the Aβ40-WT monomer and propagated using the seeding procedure described in the main text. The rows corresponding to the parallel seeded control sample (Panel *B*) exhibit a single cross peak indicative of parallel, in register β-sheet fibril structure. A corresponding antiparallel cross peak is not observed. These results indicate that the propagation procedure allows for structural conservation from parent to daughter fibrils.

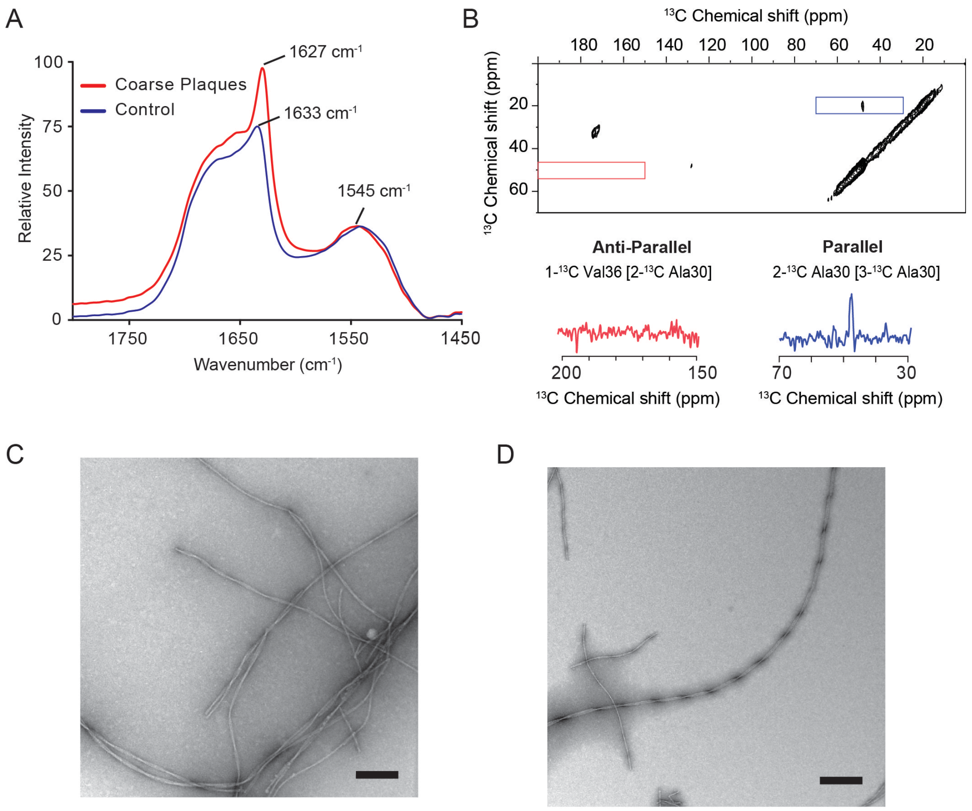

Figure S7. Parallel, in-register fibrils seeded from parenchymal amyloid obtained from an atypical AD patient. Structural studies of Aβ fibrils derived from parenchymal deposits have found that a parallel, in-register β-sheet conformation is a common feature of brain-derived Aβ fibrils. Here, we utilize parenchymal plaques isolated from an atypical AD case that presented parenchymal plaques with coarse morphology in the absence of vascular deposition. The parenchymal-derived amyloid functioned as seeds to drive fibrillization of monomeric Aβ40-WT. These fibrils allowed for a site-specific comparison between parenchymal and vascular amyloid.

The parenchymal amyloid deposits were isolated using LCM and propagated using the same seeding procedure described in the main-text (see Experimental Procedures). *A,* Overlay of FTIR spectra collected for the Aβ40-WT monomer incubated in the presence (red trace) and absence (blue trace) of parenchymal amyloid seeds. The FTIR spectrum of the sample incubated with parenchymal fibril seeds exhibits an amide I band at 1627 cm^{-1} and a shift of the amide II peak from ~1530 cm^{-1} to ~1550 cm^{-1}, demonstrating that the LCM particles seeded the formation of generation-1 fibrils. In contrast, the FTIR spectrum of the control sample exhibits a broad band between 1640 and 1690 cm^{-1}, a slight absorbance at 1633 cm^{-1}, and the absence of a shift in the amide II region. *B*, Region of 2D ^{13}C NMR spectrum of generation-3 Aβ40-WT fibrils derived from parenchymal amyloid deposits of an atypical AD case with coarse plaque

morphology using LCM. Rows are shown through the diagonal NMR resonances at 48.6 ppm for the 2-^{13}C Ala30 resonance (red) and 20.1 ppm for the 3-^{13}C Ala30 resonance (blue). Compared to the NMR spectra collected for generation-3 of vascular-derived Aβ40-WT fibrils, generation-3 parenchymal-derived Aβ40-WT fibrils are primarily composed of parallel, in-register β-sheet. These results suggest that anti-parallel β-sheet fibrils are unique to vascular amyloid. *C,D,* TEM images of generation-1 Aβ40-WT fibrils from the atypical AD case. Fibrils composed of single twisted protofilaments and pairs of twisted protofilaments were observed. The crossover distances between twists for these fibrils were typically > 100 nm. No highly twisted fibrils or short-bundled fibrils were observed. Scale bars = 140 nm (*C*), 280 nm (*D*).

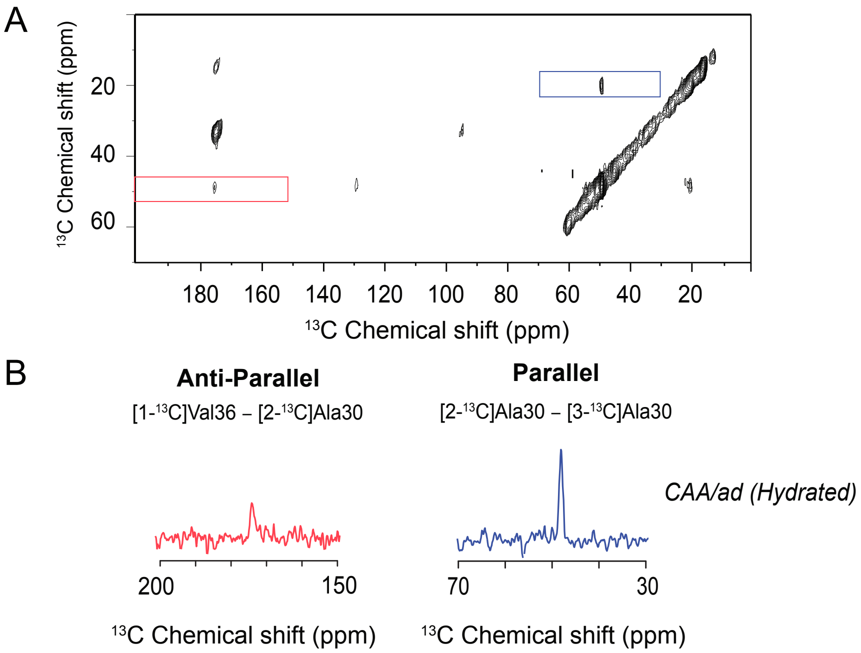

Figure S8. Presence of anti-parallel structure in fully hydrated fibrils. *A,* Region of the 2D ^{13}C NMR spectrum corresponding to generation-3 Aβ40-WT fibrils derived from the CAA/ad case using LCM. A second sample of vascular amyloid was isolated from the CAA/ad case by LCM and then propagated using the same procedure described in the main text. Upon maturation of the generation-3 sample, the sample was subject to ultracentrifugation at >100,000 x g for 2 h. The excess solvent containing potential low molecular-weight aggregates was manually removed. The remaining pellet was transferred directly into a 4 mm MAS rotor for NMR analysis. *B,* Rows are shown through the diagonal resonances of 2-^{13}C Ala30 (red) at 48.6 ppm and 3-^{13}C Ala30 (blue) at 20.1 ppm, boxed in panel (*A*), for generation-3 Aβ40-WT fibrils derived from the CAA/ad patient in the fully hydrated state. This result indicates that NMR cross peaks corresponding to anti-parallel fibril structure are observed in near-native conditions and do not arise from the presence of protofibrils or oligomers.
